# Recurrent Solitary Fibrous Tumor in a 73-Year-Old Male Presenting With Small Bowel Obstruction: A Case Report

**DOI:** 10.7759/cureus.44297

**Published:** 2023-08-28

**Authors:** Adam Talaat, Aron Mcguirt

**Affiliations:** 1 Medicine, University of Central Florida College of Medicine, Orlando, USA; 2 General Surgery, Bay Pines Veterans Affairs (VA) Health Care System, St. Petersburg, USA

**Keywords:** intra-abdominal soft tissue tumor, intra-abdominal adhesion, hemangiopericytoma, small bowel resection, solitary fibrous tumor (sft)

## Abstract

Solitary fibrous tumors (SFTs) are rare soft tissue tumors that can arise in the abdomen, pleura, and central nervous system, among other sites. Surgical resection is the mainstay of management, although recurrence rates remain substantial. This case describes a 73-year-old male treated surgically for both a recurrent SFT and small bowel obstruction (SBO) secondary to adhesions. The patient had undergone numerous intra-abdominal operations for malignant SFT since 1994, highlighting the importance of meticulous resection at the initial presentation of local disease.

## Introduction

Previously classified as hemangiopericytomas, solitary fibrous tumors (SFTs) are rare soft tissue malignancies with an age-adjusted incidence rate of about one per million persons per year [1,2]. About a third arise in the thoracic cavity, and another third arise in the abdomen; the most common intra-abdominal site is the retroperitoneum [3]. These tumors have also been described in the head and neck, central nervous system, deep soft tissues of the trunk and extremities, and bone and rarely affect superficial soft tissue [3].

Patients with intra-abdominal SFTs can be asymptomatic; they can be found incidentally on imaging or can present with symptoms of mass effect such as abdominal pain, unintentional weight loss, and obstructive symptoms [4-7]. Urinary symptoms can include urinary retention and hydronephrosis [4-6]. There are benign and malignant variations of SFTs, with malignant tumors showing more of a propensity toward recurrence and metastatic disease. The pathological features of malignant tumors include infiltrative margins, hypercellularity, necrosis, pleomorphism, and a mitotic index that is greater than 4 per 10 high-power field [8].

The prognosis of SFT is variable. One multicenter retrospective study analyzing data for 81 patients demonstrated a five-year overall survival rate of 84% for patients who underwent surgical resection with curative intent, with a worsened prognosis for malignant, metastatic, or unresectable disease [9].

En bloc surgical resection, ideally with negative margins, is the mainstay of management for all types of localized SFT [3]. Radiotherapy can play a role in adjuvant and neoadjuvant therapy, with adjuvant therapy being a widely accepted practice following the resection of high-risk central nervous system SFTs [3]. Regardless of anatomical site, studies have demonstrated decreased local recurrence rates with adjuvant or neoadjuvant radiotherapy, although survival benefit is unclear [3]. Chemotherapy is typically reserved for advanced or metastatic disease [3].

Here, we present the case of a patient who experienced SFT recurrences in the thorax, abdomen, and pelvis over the course of almost 30 years despite numerous operations and courses of adjuvant radiotherapy and chemotherapy.

## Case presentation

A 73-year-old male with a history of recurrent SFTs was admitted to the general surgery service for a small bowel obstruction (SBO). The patient had an extensive surgical history including eight operations for the resection of several solitary fibrous tumor recurrences. His first SFT resection was in 1995 for a 17 cm retroperitoneal mass, during which a part of his colon was resected. He received adjuvant chemotherapy. He underwent further resections in 1998 and 1999 for intra-abdominal and perirectal SFTs, respectively. He received adjuvant radiotherapy for the perirectal SFT in 1999.

In 2011, the patient had a recurrence with three mesenteric SFTs (one near the right lobe of the liver, one adjacent to the spleen, and one pelvic mass). Resection was performed with negative margins. Splenectomy was necessary due to one of the masses’ proximity to the splenic artery.

In 2015, seven new recurrent intra-abdominal SFTs were discovered, and resection was performed with clear margins. Again, in 2018, a computed tomography (CT) demonstrated seven intra-abdominal lesions, all of which were resected. Pathological evaluation demonstrated that the resected lesions were SFTs with negative margins. A portion of the left colon wall was also resected and closed primarily at that time.

In 2019, the patient presented with abdominal pain. A CT scan demonstrated a lesion in the right lower quadrant (RLQ) of the abdomen, as well as one in the left hemithorax near the diaphragm. A cardiothoracic surgeon resected the intrathoracic tumor with clear margins. The RLQ tumor was found to be retroperitoneal and resected from an attachment to the cecum. Pathological evaluation at that time showed oval to spindled cells with mild nuclear atypia and 5-7 mitoses per 10 high-power field, consistent with a malignant classification of SFT [8].

Separate from these countless recurrences, the patient was also diagnosed with bladder urothelial carcinoma in 2019 and bile duct adenocarcinoma in 2021. He underwent a Whipple procedure at an outside facility in 2021 and received multiple rounds of adjuvant chemotherapy (specifically, paclitaxel and gemcitabine). Comorbidities included a history of moderate to severe aortic stenosis and normocytic anemia. The patient also had a history of hypertension, which was well-controlled. Despite his medical history, the patient remained physically active.

In 2023, the patient presented to the emergency department (ED) with symptoms consistent with small bowel obstruction, specifically, obstipation of three days, bilious emesis, nausea, abdominal distention, and abdominal pain. He also complained of poorly localized, dull flank and back pain. On examination, the patient was well-appearing and in no acute distress. A cardiac examination revealed a grade II, systolic, crescendo-decrescendo murmur on auscultation at the right upper sternal border. The abdomen appeared somewhat distended. On auscultation of the abdomen, the patient had high-pitched bowel sounds. On palpation of the abdomen, there was some voluntary guarding without rigidity. The patient had mild tenderness to palpation over the right lower quadrant of the abdomen. Percussion of bilateral costovertebral angles did not elicit any tenderness. Physical examination was otherwise unremarkable.

**Figure 1:**
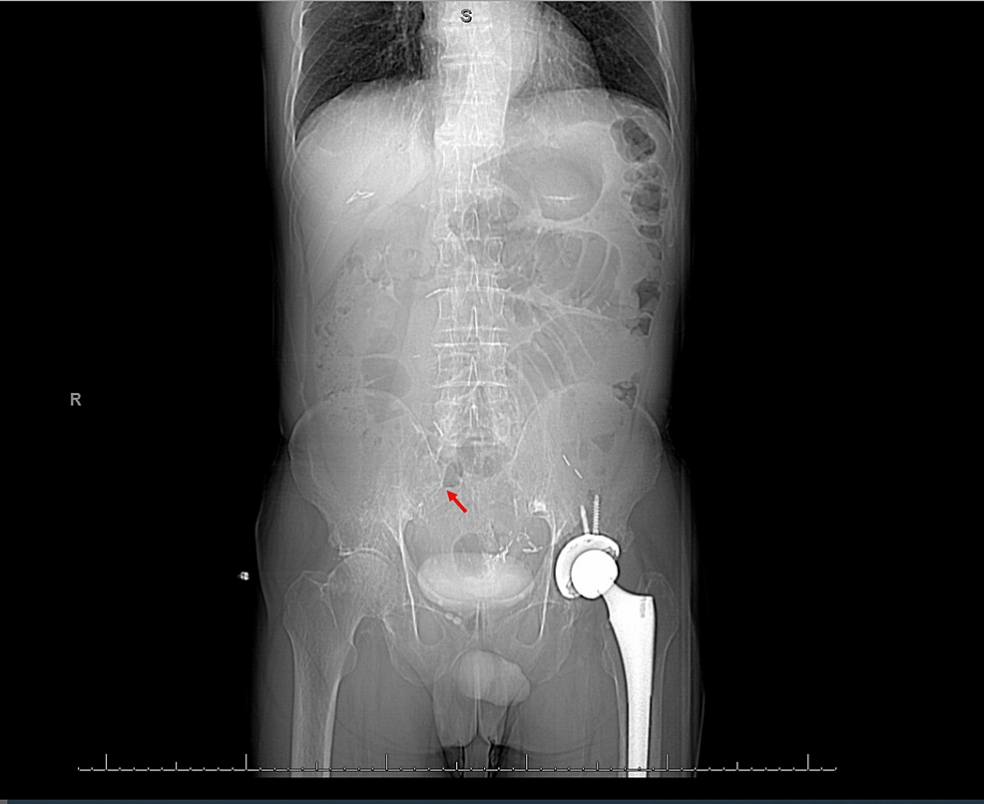
CT scan of the abdomen and pelvis without contrast (coronal view) demonstrating dilated loops of small bowel with a transition point near the terminal ileum (arrow), consistent with small bowel obstruction. CT: computed tomography

**Figure 2:**
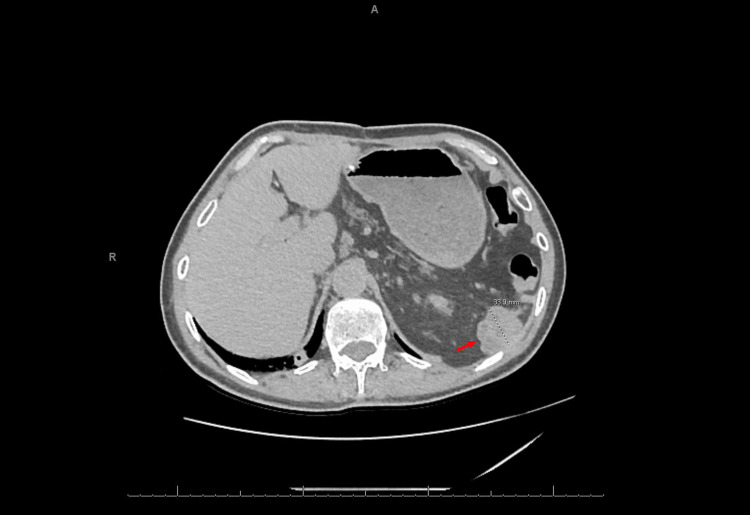
CT scan of the abdomen and pelvis without contrast (axial view) showing a lobulated mass in the splenectomy bed (arrow). Pathological evaluation of the mass demonstrated a malignant solitary fibrous tumor. CT: computed tomography

A metabolic panel was mostly unremarkable with the exception of a slight elevation in serum creatinine from the patient’s baseline. A complete blood count demonstrated anemia with hemoglobin of 9.8 g/dL and hematocrit of 30%. There was no evidence of leukocytosis. The platelet count was within normal limits. A coagulation panel demonstrated prothrombin time (PT), partial thromboplastin time (PTT), and international normalized ratio (INR) within normal limits. A non-contrast CT scan of the abdomen and pelvis demonstrated dilated loops of small bowel with a transition point near the terminal ileum (Figure 1), as well as a 3.7 cm lobulated mass in the splenectomy bed (Figure 2).

The small bowel obstruction (SBO) was initially managed conservatively with a nasogastric (NG) tube for gastrointestinal decompression, maintenance IV fluid, and nil per os (NPO) status. After three days without clinical improvement, the patient was offered surgical intervention for both his SBO and SFT recurrence. He consented to exploratory laparotomy and proceeded to surgery.

A midline vertical incision spanning from the subxiphoid to the suprapubic region was made. The fascia was divided carefully to enter the peritoneal cavity. The patient had extensive adhesive disease, especially in the RLQ. Adhesions were lysed extensively. An anastomosis near the ileocecal valve from a previous bowel resection had become adherent to the retroperitoneum, creating a kink and likely causing the obstruction. Fecalization of thick paste-like stool was noted proximal to this anastomosis, although the anastomosis itself was widely patent. Adhesions surrounding the anastomosis were carefully lysed, and the entire length of the small bowel was run several times to ensure that it was patent and without injury. In the left upper quadrant (LUQ), a mesenteric nodule was found and resected. To reach the tumor lying in the splenectomy bed, Gerota’s fascia was entered in the upper pole. The tumor was excised en bloc with the surrounding peritoneum and retroperitoneum.

From the operating room (OR), the patient was transferred to the surgical intensive care unit with an arterial line for continuous blood pressure monitoring, maintenance IV fluids, a Foley catheter to monitor fluid output, an NG tube for continued gastrointestinal decompression, and patient-controlled analgesia. The NG tube, arterial line, and Foley catheter were removed on postoperative day 2. The patient was switched to oral pain medications and transferred to the floor on postoperative day 3. On postoperative day 4, the patient began to regain bowel function with multiple bowel movements and flatulence. He was discharged on the same day.

Pathological evaluation for the LUQ mass was consistent with a malignant solitary fibrous tumor. It described a 4.5 cm solitary fibrous tumor consisting of mildly atypical small round/ovoid to spindled cells with numerous background vessels, as well as focal necrosis and cystic degeneration. The mitotic rate was at least 7 per 10 high-power fields. The mesenteric nodule was deemed a retained foreign body. The margins for the resection were negative.

The patient was followed in person at the surgery clinic two weeks after discharge. On clinic follow-up, the patient reported good appetite and bowel function. He had mild pain at the incision site but otherwise denied any abdominal or flank pain. The incision appeared to be healing appropriately without erythema or discharge. Surgical staples were removed from the incision at the same visit.

## Discussion

By far, the most common cause of small bowel obstruction (SBO) in developed countries is intra-abdominal adhesions, usually secondary to prior abdominal operations [10]. Adhesive SBO can lead to complications such as bowel ischemia or perforation, and sepsis [11]. For patients such as the one described in this case with numerous operations, adhesions are inevitable, with SBO being a foreseeable consequence. This raises the question of what could be done to prevent the recurrence of intra-abdominal SFTs, thereby minimizing the number of necessary operations.

Recurrence rates for SFT are substantial, with one meta-analysis demonstrating a range of 3%-22% recurrence for pleural SFT after surgical resection [12]. In head and neck SFT, wide margins can be difficult to achieve, and incomplete resection is associated with higher rates of recurrence and malignant transformation [13]. One site reported that no patients among a cohort of 14 with recurrent SFT were cured, despite complete salvage surgery of the first local recurrence; the researchers posited that local relapse is inevitably accompanied by dissemination [14]. All evidence appears to point to the importance of achieving negative margins on the initial resection of local SFT, regardless of the anatomical site. Our patient underwent his first surgical resection for the disease in 1995 at an outside facility, with the initial, complete pathology report unfortunately being inaccessible.

SFTs are highly vascular tumors [15]. This has prompted research into the effectiveness of antiangiogenics in SFT management. For example, a phase II clinical trial investigating the efficacy of Pazopanib among 35 patients demonstrated that this antiangiogenic agent could aid in slowing the progression of unresectable malignant and metastatic SFT [16]. Data from phase III clinical trials is lacking regarding the efficacy of antiangiogenics in decreasing recurrence rates of localized SFT when utilized in neoadjuvant or adjuvant therapy [17].

Remarkably, our patient had not only dealt with countless SFT recurrences but also bile duct adenocarcinoma and bladder urothelial carcinoma in recent years. Notably, one single-site study found that among a cohort of 82 SFT patients, 22 had experienced a secondary malignancy [18].

## Conclusions

Solitary fibrous tumors (SFTs) are rare soft tissue malignancies that can affect various anatomical locations including the abdomen. Solitary fibrous tumors have a strong propensity for recurrence despite surgical resection. All evidence appears to point to the importance of achieving negative margins on the initial resection of local SFT, regardless of the anatomical site. With almost 30 years of recurrences, complications, and additional malignancies, the patient’s experience described in this case is one of tremendous resiliency. We hope that this detailed case description will be of benefit to practitioners elsewhere as evidence continues to accumulate on how to best manage this rare disease.
